# Fluorescence Imaging of Cell Membrane Potential: From Relative Changes to Absolute Values

**DOI:** 10.3390/ijms24032435

**Published:** 2023-01-26

**Authors:** Dmitrii M. Nikolaev, Vladimir N. Mironov, Andrey A. Shtyrov, Iaroslav D. Kvashnin, Andrey S. Mereshchenko, Andrey V. Vasin, Maxim S. Panov, Mikhail N. Ryazantsev

**Affiliations:** 1 Institute of Biomedical Systems and Biotechnologies, Peter the Great Saint Petersburg Polytechnic University, 29 Polytechnicheskaya str., 195251 Saint Petersburg, Russia; 2 Nanotechnology Research and Education Centre RAS, Saint Petersburg Academic University, 8/3 Khlopina str., 194021 Saint Petersburg, Russia; 3 Institute of Chemistry, Saint Petersburg State University, 26 Universitetskii pr, 198504 Saint Petersburg, Russia; 4 Center for Biophysical Studies, Saint Petersburg State Chemical Pharmaceutical University, 14 Professor Popov str., lit. A, 197022 Saint Petersburg, Russia

**Keywords:** membrane potential imaging, cell membrane potential, potentiometric sensors, genetically-encoded voltage indicators, voltage-sensitive dyes

## Abstract

Membrane potential is a fundamental property of biological cells. Changes in membrane potential characterize a vast number of vital biological processes, such as the activity of neurons and cardiomyocytes, tumorogenesis, cell-cycle progression, etc. A common strategy to record membrane potential changes that occur in the process of interest is to utilize organic dyes or genetically-encoded voltage indicators with voltage-dependent fluorescence. Sensors are introduced into target cells, and alterations of fluorescence intensity are recorded with optical methods. Techniques that allow recording relative changes of membrane potential and do not take into account fluorescence alterations due to factors other than membrane voltage are already widely used in modern biological and biomedical studies. Such techniques have been reviewed previously in many works. However, in order to investigate a number of processes, especially long-term processes, the measured signal must be corrected to exclude the contribution from voltage-independent factors or even absolute values of cell membrane potential have to be evaluated. Techniques that enable such measurements are the subject of this review.

## 1. Introduction

Membrane potential is a fundamental physiological property of cells involved in the control of various biological processes [1]. Fast changes in membrane potential (up to hundreds of milliseconds) drive the activity of electrically excitable cells, such as neurons and cardiomyocytes. Slower changes of resting membrane potential characterize cell cycle progression [2,3], differentiation [4], insulin secretion [5], circadian firing cycles of SCN neurons [6], etc. Decrease of resting membrane potential induces cell proliferation and is related to cancer progression [7,8] as well as other disorders [9,10,11]. Therefore, the development of tools and techniques for membrane potential measurements is an important prerequisite for further progress in many areas of cell biology, biosensing, medicine, pharmacology, etc.

Electrophysiological methods provide accurate voltage measurements but possess several critical drawbacks. Injury of the membrane by microelectrodes during an experiment can alter membrane characteristics and become a non-negligible source of error [12,13]. The number of cells measured simultaneously is limited to a single-cell (patch-clamp) or hundreds of cells (multi-electrode arrays) with relatively poor spatial resolution [14]. Besides, electrophysiological methods require complicated preparation procedures, especially for in vivo measurements, and are challenging to use in long-term studies [15].

An alternative approach is membrane potential imaging, which utilizes methods to monitor voltage-dependent changes of the optical signal detected either from label-free cells [16,17,18], or from molecular, biomolecular or nanoparticle-based potentiometric sensors introduced into cells. Currently, the techniques based on different types of optical signals have been developed, including fluorescence, light scattering, transmitted light intensity, birefringence, second harmonic signals, etc. [18,19]. Voltage imaging does not have a limit on the number of analyzed cells, ranging from single-cell measurements to large cell populations. Spatial and temporal resolutions of voltage imaging depend on the applied sensor, and currently available toolkit provides sensors and methods to perform measurements with subcellular or single-cell spatial resolutions and investigate processes starting from millisecond timescale, which is typical for neuronal or cardiomyocyte action potentials, to days and weeks, which is an important timescale to study pathological processes.

The majority of membrane potential imaging studies record voltage-induced alterations of fluorescence intensity detected from the potentiometric sensors located in cells. This approach reports the relative changes of membrane potential and for a large number of biological processes was shown to provide robust results. For example, cell membrane potential imaging has been successfully applied in biological and biomedical research for the investigation of brain functioning [20,21,22,23,24], development of the vertebrate nervous system [25], cardiac electrical activity [26,27,28], cell development [29,30,31], etc. However, the detected fluorescence intensity alterations are associated not only with voltage changes, but also with a number of voltage-independent processes, such as cell motion or intracellular transport of macromolecules. When the value of voltage-induced fluorescence changes is much larger than the effect of non-target processes, the magnitude of error will be modest and can be neglected. Otherwise, specific techniques have to be applied to eliminate the contribution of voltage-independent factors from the detected fluorescence signal. Besides, to obtain quantitative results from membrane potential imaging experiments, the correspondence between the magnitude of detected optical signal and voltage values have to be derived using calibration techniques.

Techniques for membrane potential imaging, in which the contribution from voltage-independent factors into the detected optical signal is not taken into account, have been already covered in a number of reviews [32,33,34,35,36,37,38,39]. These studies reviewed classes of available sensors, principles of functioning, measurement methods, range of applicability, etc. Here, we rather focus on the techniques that provide the possibility to eliminate contributions in fluorescence signal that occur from factors other than voltage and methods that are applied to derive absolute cell membrane potential values. The two most common classes of sensors for membrane voltage imaging, fluorescent organic dyes and fluorescent genetically-encoded voltage indicators, will be considered.

The review is organized as follows. In Section 2, we will briefly describe two widely used approaches for membrane potential imaging: recording the intensity of fluorescence and measurement of excited state lifetime. In Section 3, we will describe the most commonly used types of molecular and biomolecular sensors. In Section 4, we will cover the techniques used to eliminate fluorescence changes caused by voltage-independent factors and perform reliable measurements of cell membrane potential changes in different processes. Finally, in Section 5 we will consider calibration techniques that allow deriving absolute values of cell membrane potential.

## 2. Two Most Common Fluorescence Techniques for Membrane Potential Imaging

Two physical properties affected by voltage are most commonly used to record membrane potential: fluorescence intensity and excited state lifetime.

*Fluorescence intensity* can reflect the relative changes of membrane potential, but this property is also subjected to a number of voltage-independent factors (see Section 4). Measurements can be performed with standard fluorescence or confocal microscope equipped with a digital camera [40].

*Excited state lifetime*, the time between absorption of excitation photon by the fluorophore and the re-emission of fluorescence photon, can be expressed as follows:

(1) $$\tau_{exc}=\frac{1}{k_{fl}+k_{nr}}$$

Here, $k_{fl}$ is the rate constant for fluorescence, $k_{nr}$ is the rate constant for non-radiative decay. For several sensors, linear voltage-dependence of $\tau_{exc}$ was demonstrated [41,42]. Measurements of excited state lifetime can be performed with equipment for time-resolved fluorescence microscopy [43].

Membrane voltage can affect both fluorescence intensity and excited state lifetime, and measurement of these properties is widely used for membrane potential imaging. To date, several classes of sensors with different functioning principles have been developed. Sensors with linear voltage-dependence of fluorescence properties are preferred for obtaining unambiguous correspondence between detected optical signal and membrane voltage.

## 3. Types of Molecular and Biomolecular Potentiometric Sensors

The currently available toolkit for membrane potential imaging contains a large variety of molecular and biomolecular sensors with voltage-dependent fluorescence. These sensors can be composed of a single molecule or include several components. Below we follow one of the commonly-used classifications of molecular and biomolecular sensors.

### 3.1. Electrochromic Organic Dyes

**Figure 1:**
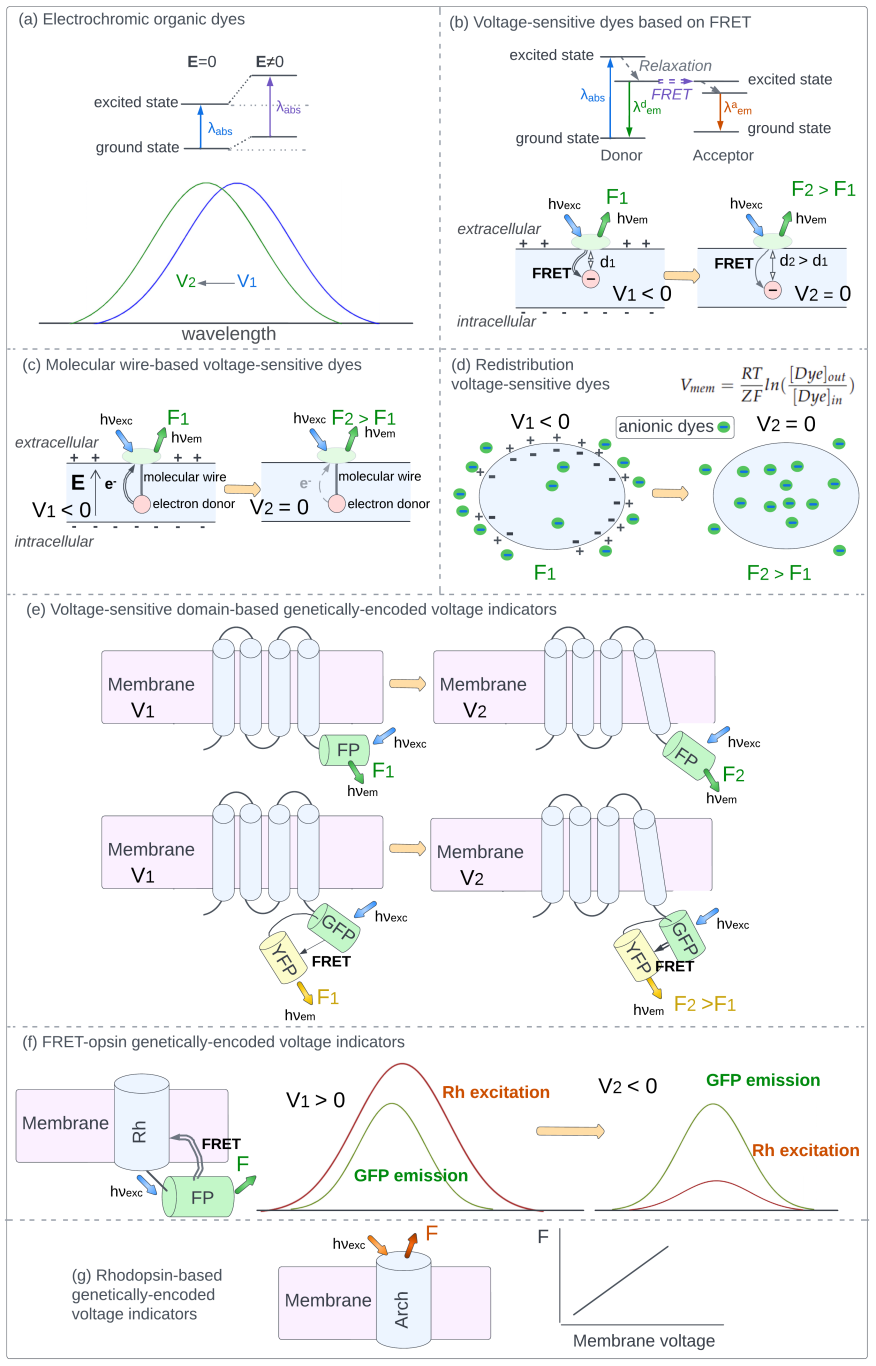
Commonly-used classes of molecular and biomolecular potentiometric sensors. (**a**) Electrochromic organic dyes. *Upper.* Electrochromic organic dyes are characterized by different electron distributions in the ground and excited states. Interaction with an external electric field **E** alters the energies of the ground and excited states to a different extent, resulting in the shift of absorption, excitation and emission bands. *Lower*. Spectral shift of electrochromic dye upon membrane voltage change can be utilized for membrane potential imaging. The depicted band can represent absorption, excitation, or emission spectral band. (**b**) FRET-based organic dyes. *Upper.* Fluorescence resonance energy transfer (FRET) can occur between two molecules (donor and acceptor) with overlapping excitation and emission bands. After photoexcitation, the donor can either re-emit a photon or transfer energy to the acceptor via FRET. If the acceptor is fluorescent, it can emit a photon with a longer wavelength. *Lower.* FRET pair includes an immobile donor fluorophore (green oval) attached to the outer surface of the cell membrane and a mobile lipophilic ion (acceptor) located inside the membrane (red circle). The fluorescence of the donor is quenched via FRET. Upon depolarization the acceptor moves further from the donor, resulting in the decrease of FRET efficiency and an increase in donor fluorescence. (**c**) Molecular wire-based voltage-sensitive dyes. A fluorophore (green oval) is attached to the outer surface of the cell membrane. Its fluorescence is quenched by electron transfer from an electron donor (red circle) through a molecular wire. Depolarization decreases the rate of electron transfer, resulting in the enhancement of fluorescence. (**d**) Redistribution voltage-sensitive dyes. At equilibrium the ratio of extracellular and intracellular concentrations of charged membrane-permeable dyes is determined by membrane potential in accordance with the Nernst equation. Upon depolarization the intracellular concentration of anionic dyes (green circles) increases leading to the enhancement of fluorescence detected from the cell interior. (**e**) Voltage-sensitive domain-based genetically-encoded voltage indicators. *Upper.* Voltage-induced structural reorganization of a voltage-sensitive domain (VSD, blue cylinders) is passed to the voltage-independent fluorescent protein (FP, green cylinder) through a peptide linker. The fluorescence of the construct is voltage-dependent. *Lower* Two FPs with overlapping excitation and emission bands are attached to the VSD. Voltage-induced structural reorganization of VSD changes the relative position of FPs, resulting in the change of FRET efficiency and, therefore, fluorescence intensities of both donor and acceptor. (**f**) FRET-opsin genetically-encoded voltage indicators. Fluorescent protein (FP) is attached to microbial rhodopsin (Rh). At positive voltages the chromophore of Rh is protonated and its absorption efficiently quenches FP emission via FRET. Upon depolarization, the concentration of Rh with protonated chromophore decreases, leading to the lowering of the red-shifted absorption band, decrease of FRET efficiency, and the enhancement of detected FP fluorescence. (**g**) Rhodopsin-based genetically-encoded voltage indicators. Several microbial rhodopsins, such as archaerhodopsin-3 (Arch), demonstrate intrinsic linear voltage dependence of fluorescence intensity.

Electrochromic dyes are characterized by a shift of absorption and fluorescence spectra in an external electric field (Stark effect). Photoexcitation of electrochromic dyes induces intramolecular charge transfer resulting in different electron distributions in the ground and excited states. Interaction with an external electric field alters the energies of the ground and excited states to a different extent, and the shift of absorption/emission bands is observed (Figure 1a). A number of methods utilizing electrochromic dyes have been developed for membrane potential imaging. These methods are based on monitoring the changes of absorption/emission intensity at the selected wavelength, monitoring the changes of total fluorescence excited at the selected wavelength, as well as methods based on two-photon absorption and second harmonic generation, as described in a number of comprehensive reviews [33,34,44,45,46,47,48].

The main advantage of electrochromic dyes is the ultrafast kinetics of fluorescence response to voltage change, which enables researchers to monitor processes with submillisecond temporal resolution. On the other hand, due to small magnitudes of voltage-induced spectral shifts, around 10 nm per 100 mV, electrochromic dyes do not demonstrate high voltage sensitivity. Usually, the magnitude of fractional change of fluorescence intensity (ΔF/F) per 100 mV lies in the 10–20% range, limiting the accuracy of measurements [33,34,49,50].

### 3.2. Voltage-Sensitive Dyes Based on FRET

Fluorescence resonance energy transfer (FRET) is a process that can occur between two light-sensitive molecules, if the emission band of the first molecule (donor) and the absorption band of the second molecule (acceptor) overlap (Figure 1b). Emission of the donor fluorophore is absorbed by the acceptor via a non-radiative energy transfer, and the efficiency of this transition is proportional to the inverse sixth power of the distance between the donor and the acceptor. Two types of FRET-based sensors are most widely applied. In the sensors of the first type, the acceptor is not fluorescent. Therefore, in experiment only the donor fluorescence can be detected, which enhances when the distance between the donor and acceptor increases. In the sensors of the second type, both components are fluorescent and changes in donor fluorescence, acceptor fluorescence, or both signals can be monitored.

Usually, the donor is attached to the extracellular surface of the cell membrane, and its position does not change in the experiment. The negatively charged acceptor is placed inside the plasma membrane and moves between extracellular and intracellular membrane surfaces in response to voltage changes. Therefore, the change in the membrane potential leads to an increase or decrease in the distance between the donor and the acceptor. In such systems, FRET efficiency and, accordingly, the detected fluorescence intensity become potential-dependent (Figure 1b).

While early variants of FRET-based sensors had response times lasting several milliseconds [51,52], more recent examples provide sub-millisecond temporal resolution [53,54,55]. An advantage of FRET-based dyes is the high voltage sensitivity with fractional fluorescence change (ΔF/F) ranging from dozens to hundreds of percent per 100 mV [56,57]. A general obstacle related to sensors of this type is the necessity to accurately tune donor and acceptor concentrations. More information about the available FRET-based dyes and their applications in membrane potential imaging can be found in a number of reviews [33,57,58,59,60].

### 3.3. Molecular Wire-Based Voltage-Sensitive Dyes

Molecular wire-based dyes are two-component systems consisting of a fluorophore and an electron-rich moiety (electron donor) connected with a molecular wire. After photoexcitation fluorescence emission and electron transfer from the donor become competing processes that convert fluorophore back to the ground state. The level of fluorescence quenching is determined by the rate of electron transfer. In an external electric field the rate of electron transfer depends on the magnitude of the field as well as the angle between the molecular wire and the electric field with the largest voltage-sensitivity observed when the wire is oriented parallel to the electric field [61].

This type of construct was implemented in VoltageFluors, a recently developed [pclass of dyes [62]. The fluorophore is attached to the outer surface of the cell membrane, an electron-rich moiety is located inside the lipid bilayer, and the connecting molecular wire is oriented perpendicular to the membrane surface, therefore, parallel to the membrane electric field (Figure 1c). In such a system, cell membrane potential efficiently alters the rate of electron transfer along the molecular wire and, therefore, the fluorescence intensity detected from the fluorophore.

The main advantage of sensors based on photoinduced electron transfer is the ultrafast response rates of fluorescence, which lie in the range of dozens of nanoseconds [63] and provide the ability to record the fastest processes in neurons and cardiomyocytes [64]. The most recent sensors also demonstrate high voltage sensitivity with relative change of fluorescence intensity exceeding 60% per 100 mV [65,66,67]. Recent works have extensively reviewed molecular wire-based dyes considering the sensors developed to date and their applications [33,35,63,68].

### 3.4. Redistribution Voltage-Sensitive Dyes

Membrane-permeable charged dyes placed in the vicinity of a cell with non-zero membrane potential will move between the cell interior and extracellular medium until the electrochemical equilibrium is established. At equilibrium the ratio of extracellular and intracellular dye concentrations is determined by cell membrane potential in accordance with the Nernst equation:

(2) $$V_{mem}=\frac{RT}{ZF}ln\left(\frac{{\left[Dye\right]}_{out}}{{\left[Dye\right]}_{in}}\right)$$

Here, $V_{mem}$ is cell membrane potential, R is the universal gas constant, T is the absolute temperature, Z is the charge of the dye, F is the Faraday constant, ${\left[Dye\right]}_{out}$ and ${\left[Dye\right]}_{in}$ are the extracellular and intracellular concentrations of a charged dye, respectively. Therefore, if the relation between detected fluorescence intensity and dye concentration is established, cell membrane potential can be directly evaluated from the fluorescence measurements (Figure 1d). The fluorescence intensity of dye molecules located in the cell interior can be detected with the flow cytometry technique [69]. To evaluate extracellular dye concentration fluorescence from the intracellular region is measured after complete depolarization of cells (zero membrane potential), i.e., when extracellular and intracellular dye concentrations are equal. Such depolarization can be achieved by adding ionophores that form pores in the membrane, such as paraformaldehyde [70] or gramicidin [71]. For anionic dyes, usually oxonols [72], intracellular concentration is low in the normal state of a cell (negative membrane potential) and becomes higher upon cell depolarization, i.e., when cell membrane potential is increased. Cationic dyes, usually carbocyanines or rhodamine derivatives, demonstrate the reverse trend [29,73]. The main advantage of redistribution dyes is the high magnitudes of fractional fluorescence change in response to voltage. On the other hand, the response times are limited to the timescale of seconds and minutes required for dye molecules to penetrate the membrane. Therefore, cationic and anionic fluorescent dyes are used only to monitor potential changes in long-term processes, such as cell differentiation [29,69,74]. More information about available redistribution dyes and their applications for membrane potential imaging can be found in a number of works [68,73,75,76].

### 3.5. Voltage-Sensitive Domain-Based Genetically-Encoded Voltage Indicators

Sensors constructed as a fusion of proteins with voltage-independent fluorescence and non-fluorescent transmembrane proteins or protein domains that undergo structural reorganization upon voltage change were shown to possess voltage-dependent fluorescence (Figure 1e). Conformational changes in the voltage-sensitive domain induced by membrane depolarization or hyperpolarization are passed to the fluorescent protein via a peptide linker, leading to the change of the fluorophore local environment and the change of fluorescence intensity. Fluorescence voltage dependence properties were shown to be determined by the nature of the fluorescent protein and the voltage-sensitive domain, the length of the peptide linker, and the position for the fusion of two components [77,78,79,80].

Several sensors were constructed as a fusion of two fluorescent proteins with overlapping emission/absorption bands to the same voltage-sensitive domain. For such sensors structural reorganization of the domain alters the relative position of fluorescent proteins, therefore, the level of FRET between them (Figure 1e). The fluorescence intensities of the donor and acceptor become voltage-dependent and both signals can be used to monitor membrane potential changes [81,82]. The currently available sensors from this class demonstrate relative changes of fluorescence signal up to ∼50% per 100 mV and the response times in the range of several milliseconds [77,78,79,80,82]. Up to date, a large variety of voltage-sensitive domain-based genetically-encoded voltage indicators have been developed, and their spectral properties, voltage-dependence characteristics and applicability were the subject of many reviews [83,84,85,86,87,88,89].

### 3.6. FRET-Opsin Genetically-Encoded Voltage Indicators

Microbial rhodopsins are transmembrane proteins that demonstrate a large blue shift of absorption band upon decrease of cell membrane potential, which is associated with voltage-induced chromophore deprotonation. At positive voltage values absorption band of microbial rhodopsins largely overlap with the emission band of blue/green/yellow fluorescent proteins. Therefore, at positive voltages the emission of a fluorescent protein attached to non-fluorescent microbial rhodopsin will be effectively absorbed by the rhodopsin via FRET, resulting in the low intensity of the detected fluorescence signal. Voltage decrease is accompanied by the lowering of the red-shifted absorption band attributed to rhodopsins with protonated chromophore, resulting in the decrease of FRET efficiency and the enhancement of detected fluorescence (Figure 1f). A few examples were reported for this type of sensor with relative changes of fluorescence intensity up to 18% per 100 mV and the response times ranging from <1 ms to ∼7 ms [90,91,92].

### 3.7. Rhodopsin-Based Genetically-Encoded Voltage Indicators

Several proteins from the family of microbial rhodopsins, such as archaerhodopsin-3 and its mutants, possess the intrinsic voltage dependence of fluorescence intensity (Figure 1g) [93,94]. The mechanism of fluorescence voltage-dependence is still not clear, even though several studies on this problem have been reported [95,96]. Up to date, a large number of rhodopsin-based sensors have been developed using a directed evolution approach, which does not require detailed knowledge of underlying mechanisms [97,98,99]. The main advantages of sensors from this class are the fast response rates, up to submillisecond temporal resolution, high sensitivity of fluorescence signal with the fractional change of fluorescence intensity up to 90% per 100 mV, and the ability to vary absorption band maxima values in a wide range [97,98,99,100,101,102,103,104]. The main drawback limiting the application of sensors from this class is the dimness of the detected fluorescence. Fluorescence quantum yields of currently available rhodopsin-based sensors are in the range 0.8–1.2% [83,99,105]. Recent reviews have covered the currently available rhodopsin-based sensors focusing on their spectral properties and applicability for membrane potential imaging [83,84,87].

## 4. Elimination of Fluorescence Changes Caused by Factors Other than Voltage

The fluorescence signal recorded in membrane potential imaging experiments is affected by a number of concomitant processes besides membrane potential alterations. When voltage-induced changes of fluorescence signal are much larger than the effect of side processes, the latter can be neglected. For such processes, robust results can be obtained by monitoring voltage-dependent changes of fluorescence intensity with a standard fluorescence or confocal microscope. However, in a large number of biological processes voltage-independent side factors altering fluorescence intensity become a significant source of error. Investigation of such processes with membrane potential imaging technique requires the application of methods that eliminate fluorescence changes caused by factors other than voltage.

The most frequent source of error in cell membrane potential imaging experiments is the change in the local concentration of sensors that occurs during the target process. Concentration changes can be a result of different factors, including redistribution of motile organic dyes, the motion of cells as in the case of contracting cardiomyocytes, intracellular transport of macromolecules, etc. This problem is particularly acute for long-term processes when the above-mentioned factor becomes non-negligible [28,41,106], and for in vivo studies due to sample movement relative to the camera caused by animal motion, breathing or other physiological processes [27,107]. Besides, the local concentration of fluorescent sensors decreases with time due to the bleaching of fluorophores. The rate of bleaching is not equal for all fluorophores and depends not only on the nature of the fluorophore, but also on the intensity of illumination and on the variation of the local environment, which often cannot be controlled in experiment [31,42].

Concentration dependence of optical signals can be eliminated by a synchronous recording of two or more separable optical signals. Then one can derive a function of these signals that allows to cancel out concentration-dependence but preserve voltage-dependence at the same time. The most simple such function is the ratio of two signals possessing different voltage dependence (ratio-based approaches). A possible alternative is to record a voltage-dependent property that does not depend on sensors concentration, such as excited state lifetime.

In addition to concentration changes, detected optical signal can be altered by the changes in the local environment of sensors that occur during the experiment. These factors include the alterations in ionic strength, pH, viscosity, temperature, lipid composition [28,40,41,108,109], as well as the movement of macromolecules that bind potentiometric organic dyes [110,111,112]. Protocols that eliminate optical signal changes caused by environmental factors can be developed for some systems, but require detailed knowledge of how these factors alter the detected signal and how the potentiometric sensor works.

Finally, a source of error during membrane potential imaging experiments comes from instability in illumination intensity, the sensitivity of the detector, and other possible equipment-related measurement errors. This factor can be scaled down with the appropriate software and will not be considered here since it was described in a recent comprehensive review [40].

### 4.1. Eliminating Concentration-Dependence of Detected Fluorescence with Ratio-Based Approaches

**Figure 2:**
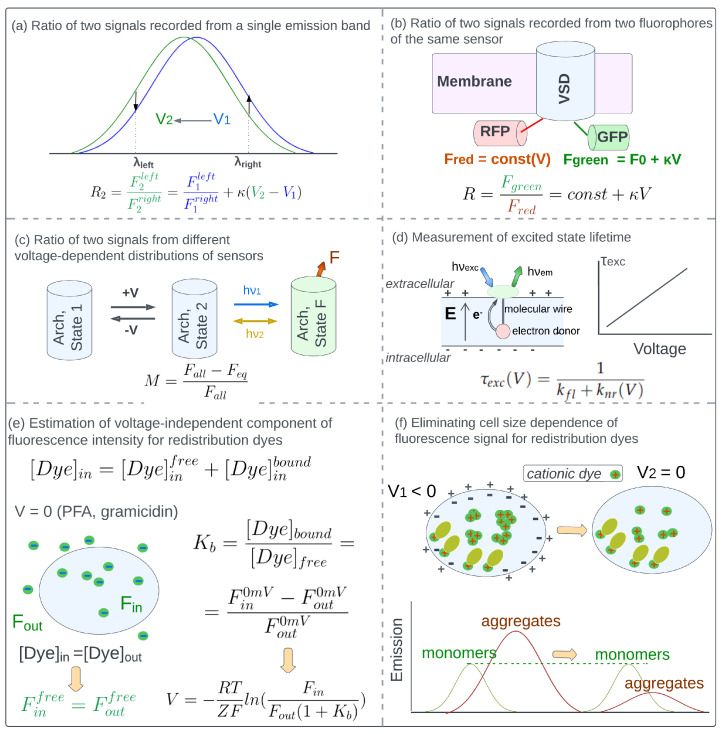
Elimination of fluorescence changes caused by factors other than voltage. (**a**) Ratio of two signals recorded from a single emission band. The voltage-induced shift of the emission band of electrochromic dyes results in the opposite changes of emission intensities corresponding to wavelengths at the left and right wings of the band. The ratio of fluorescence intensities at two wavelengths from the left and right wings of the emission band does not depend on sensors concentration and was shown to be linearly dependent on voltage [31,107]. (**b**) Ratio of two signals recorded from two fluorophores of the same sensor. Sensor is constructed as a fusion of a voltage-sensitive domain (VSD) and two fluorescent proteins—green (GFP) and red (RFP). Voltage-dependent structural reorganization of VSD is passed to FPs, resulting in linear fluorescence voltage-dependence of GFP. The fluorescence intensity of RFP remains voltage-independent. The ratio of two signals can be used to cancel out concentration dependence [28,106]. (**c**) Ratio of two signals from different voltage-dependent distributions of sensors. Microbial rhodopsin Arch D95H demonstrates linear fluorescence response to membrane potential changes attributed to the voltage-dependent equilibrium between the fluorescent (F) and non-fluorescent states of the protein [114]. In the proposed protocol the whole population of sensors was converted into state F with blue light illumination and the corresponding fluorescence intensity $F_{all}$ was measured. Afterward, orange light was used to establish an equilibrium with voltage-dependent concentration of state F, and the fluorescence intensity $F_{eq}$ was measured. The relative decrease of fluorescence intensity was shown to be a robust concentration-corrected metrics. (**d**) Excited state lifetime can be used for membrane potential imaging. This property was shown to be independent of fluorophore bleaching and illumination conditions, largely independent of fluorophore concentration. Voltage may affect the excited state lifetime by altering the rate constant of non-radiative decay ($k_{nr}$) [41]. (**e**) Estimation of voltage-independent component of fluorescence intensity for redistribution dyes. The fluorescence intensity detected from the cell interior is determined by the intracellular dye concentration, which should be directly related to membrane potential value in accordance with the Nernst equation. However, a part of dye molecules are bound to intracellular interaction sites and provide equal contribution to detected fluorescence at any membrane potential value. To determine the binding constant $K_{b}$ and derive the corrected equation for estimating membrane potential the difference in fluorescence intensity detected from cell interior $F_{in}$ and extracellular medium $F_{out}$ at 0 mV was measured [115]. (**f**) Eliminating cell size dependence of fluorescence signal for redistribution dyes. At high concentrations cationic dyes demonstrate two emission bands—a green band attributed to dye monomers and a red-shifted band attributed to dye aggregates. At saturating concentration all intracellular interaction sites are occupied at any voltage by dye monomers and green fluorescence is not affected by voltage changes. Upon depolarization the concentration of intracellular dye aggregates decreases, resulting in the decrease of red signal. Both green and red signals are proportional to cell size and their ratio can be used to cancel out cell size dependence [116].

*Ratio of two signals recorded from a single emission band.* The voltage-induced shift of the emission band of electrochromic dyes results in the opposite changes of emission intensities corresponding to wavelengths at the left and right wings of the band (Figure 2a). The ratio of fluorescence intensities at two wavelengths from the left and right wings of the emission band does not depend on sensor concentration and remains voltage-dependent. The two emission wavelengths are selected to obtain the ratio with linear voltage dependence and the highest possible voltage sensitivity. The strategy allowed eliminating the effect of sensors concentration changes caused by the motion of arterioles during vascular responses [107] and the motion of epicardium during cell membrane potential imaging in isolated hearts [31]. However, the detected ratios showed baseline drifts caused by different photobleaching rates of signals from the low and high wavelength wings of emission band [31]. An alternative approach that can be used with electrochromic dyes applies the excitation of sensors at two wavelengths and the detection of total fluorescence. Voltage-induced shift of absorption band results in the opposite changes of absorption intensities at the left and right wings of the band and, therefore, opposite changes of total fluorescence excited at these two wavelengths [109,113].

*Ratio of two signals recorded from two fluorophores which are parts of a single sensor.* Two signals can be obtained from two fluorophores of a single sensor. This approach can be applied if two requirements are fulfilled: the emission bands of fluorophores should not overlap and they should possess different voltage-dependence. Besides, the method can provide robust results when the bleaching rates of two fluorophores are identical and environmental factors have the same effect on both fluorophores. The approach is applicable for FRET-based sensors with both donor and acceptor fluorescent. For sensors of this type, the voltage sensitivity of ratio is a sum of voltage sensitivities of donor and acceptor fluorescence. The method was applied to cancel out motion-induced changes in the detected optical signal and investigate the electrical activity of several eukaryotic cells, including astrocytoma cells and beating cardiac myocytes [51,52].

The approach was also applied for sensors constructed as the fusion of two fluorescent proteins (green and red) to the voltage-sensitive domain (Figure 2b). Voltage-induced reorganization of the domain was passed to both proteins via peptide linkers, altering the emission intensity of the green fluorescent protein, but keeping the emission intensity of the red fluorescent protein intact [106]. The ratio of green and red signals allowed the elimination of the concentration-related signal changes caused by the contraction of cardiomyocytes and the motion of cells during cell-cycle studies [28]. For HEK293 cells, the method allowed monitoring changes in resting membrane voltage caused by ectopic expression of potassium channels on the timescale of several days. However, a large cell-to-cell variation of detected signal prevented the measurement of cell membrane potential changes with single-cell resolution [106].

*Ratio of two signals from different voltage-dependent distributions of sensors.* Microbial rhodopsin Arch D95H demonstrates linear fluorescence response to cell membrane potential changes attributed to the voltage-dependent equilibrium between the fluorescent (F) and non-fluorescent states of the protein [114]. The fluorescence intensity of state F was supposed to be independent of the voltage, therefore, fluorescence response to membrane potential change was attributed only to the change in the concentration of state F. This concentration was estimated using the following protocol [114]. The whole population of proteins was converted into state F with blue light illumination and the corresponding fluorescence intensity $F_{all}$ was measured (Figure 2c). Afterward, orange light was used to establish an equilibrium with voltage-dependent concentration of state F, and the emission intensity $F_{eq}$ was measured. The relative decrease of fluorescence intensity upon equilibrium establishment was shown to be a robust concentration-corrected metric for membrane potential measurements:

(3) $$M=\frac{F_{all}-F_{eq}}{F_{all}},$$

The method was tested on HEK cells and allowed deriving the value of resting cell membrane potential with a 10 mV resolution [114].

### 4.2. Measurement of Excited State Lifetime

A possible approach to avoid signal components related to the alteration of sensor concentration is to measure excited state lifetime rather than fluorescence intensity. Excited state lifetime was shown to be independent of fluorophore bleaching and illumination conditions, and largely independent of fluorophores concentration [43]. Environmental factors, including voltage, may affect excited state lifetime by changing the rate constant of non-radiative decay ($k_{nr}$), and for a few sensors $k_{nr}$ and, therefore, $\tau_{exc}$ were shown to possess linear voltage-dependence:

(4) $$\tau_{exc}\left(V\right)=\frac{1}{k_{fl}+k_{nr}\left(V\right)}$$

It should be noted that due to the complex nature of non-radiative decay, voltage-dependence of fluorescence intensity does not guarantee the voltage-dependence of excited state lifetime [42,43]. Besides, the accuracy of the method largely depends on the magnitude of $\tau_{exc}$ changes caused by other environmental factors during membrane potential imaging experiments.

Linear voltage dependence of $\tau_{exc}$ was demonstrated for molecular wire-based organic dyes [41] (Figure 2d). For these dyes, $k_{nr}$ is determined by the rate of electron transfer from the electron donor to the fluorophore molecule upon photoexcitation, which is linearly dependent on voltage. Measurement of excited state lifetime allowed monitoring slow voltage transients upon stimulation of HEK cells with growth factor providing a 5 mV voltage resolution [41]. For two sensors constructed as fluorescent proteins fused to a voltage-sensitive domain, the excited state lifetime was also found to be linearly dependent on external electric field [42], but provided limited voltage resolution due to the large contribution to $\tau_{exc}$ from environmental factors other than voltage.

### 4.3. Eliminating the Effect of Side Factors Other than Sensors Concentration Changes

Besides voltage and concentration, fluorescence intensity of potentiometric sensors can be affected by environmental factors, including the local composition of lipids and macromolecules, ionic strength, viscosity, etc. [28,40,41,108,109]. Elimination of these effects can be performed for some systems0 if the nature of the effect, as well as the principle of functioning of the sensor, is known. Here we provide two specific protocols developed for redistribution dyes.

The fluorescence intensity of redistribution dyes detected from the cell interior is determined by the intracellular dye concentration, which should be directly related to cell membrane potential value in accordance with the Nernst Equation (Equation (2)). However, a part of dye molecules are bound to intracellular interaction sites (macromolecules and organelles), and provide equal contribution to detectung fluorescence at any membrane potential value. The effect of bound dyes can be removed if the binding constant $K_{b}$ is determined. The protocol to eliminate the contribution from bound molecules was developed for dyes with linear concentration dependence of fluorescence intensity, i.e., when Equation (2) can be transformed into the following relationship [115]:

(5) $$V_{mem}=\frac{RT}{ZF}ln\left(\frac{F_{out}}{F_{in}}\right),$$

where $F_{out}$ and $F_{in}$ are fluorescence intensities detected from dye molecules located in the extracellular medium and cell interior, respectively. Binding constant $K_{b}$ was derived from the difference between $F_{in}$ and $F_{out}$ and 0 mV (Figure 2e):

(6) $$K_{b}=\frac{{\left[Dye\right]}_{bound}}{{\left[Dye\right]}_{free}}=\frac{F_{in}^{0mV}-F_{out}^{0mV}}{F_{out}^{0mV}}$$

Here, $F_{in}^{0mV}$ and $F_{out}^{0mV}$ are fluorescence intensities measured for completely depolarized cells from the cell interior and extracellular medium, respectively. Then the following corrected equation was used to derive membrane voltage from fluorescence intensity measurements:

(7) $$V_{mem}=\frac{RT}{ZF}ln\left(\frac{F_{out}^{free}}{F_{in}^{free}}\right)=-\frac{RT}{ZF}ln\left(\frac{F_{in}}{F_{out}\left(1+K_{b}\right)}\right)$$

The method was successfully applied to investigate the resting membrane potential of several cell lines [115].

Another problem associated with the application of redistribution dyes for membrane voltage imaging is associated with the cell-to-cell variation of cell size as well as changes in cell size that can occur during the target process. According to the Nernst equation, cell membrane potential can be estimated from the intracellular dye concentration, but the detected fluorescence is rather proportional to the total amount of dye molecules in the cell interior. To provide robust results the detected fluorescence has to be divided by parameters related to the cell size. The protocol to eliminate cell size dependence was developed for cationic organic dyes, which are characterized by the existence of two emission bands at high concentrations (Figure 2f). The green band is attributed to dye monomers, while the red-shifted band is attributed to dye aggregates. These two signals were detected from prokaryotic cells stained with a cationic dye at a high concentration. The green signal was emitted by dye monomers bound to intracellular interaction sites. At the saturation concentration used in the experiment (30 μM), its intensity was voltage-insensitive since all interaction sites were occupied at any membrane potential value. On the other hand, the magnitude of the red signal demonstrated voltage-dependence in agreement with the Nernst equation—the concentration of intracellular dye aggregates decreased upon membrane depolarization. Both green and red signals were proportional to cell size and their ratio was successfully applied to cancel out cell size dependence. The method was used to monitor membrane potential changes that occurred after the addition of depolarizing agents into cell medium on the timescale of several minutes [116]. However, the application of cationic dye at the high concentration required for the experiment is related to high toxicity and can affect the target biological processes.

## 5. Calibration Techniques That Are Applied to Derive the Correspondence between the Detected Optical Signal and Membrane Voltage Values. Measurement of Absolute Values of Cell Membrane Potential

Measurement of absolute cell membrane potential values requires the knowledge of correspondence between the magnitudes of the optical signal detected from potentiometric sensors and membrane potential values. This correspondence is obtained by measuring optical signal magnitudes for a series of determined voltage values and plotting a calibration curve. Since voltage-dependence characteristics of the optical signal of potentiometric sensors are sensitive to different factors including cell type, type of sample (cell culture, tissue, in vivo measurements), experimental conditions, calibration has to be performed for each system of interest.

### 5.1. Patch-Clamp Technique

**Figure 3:**
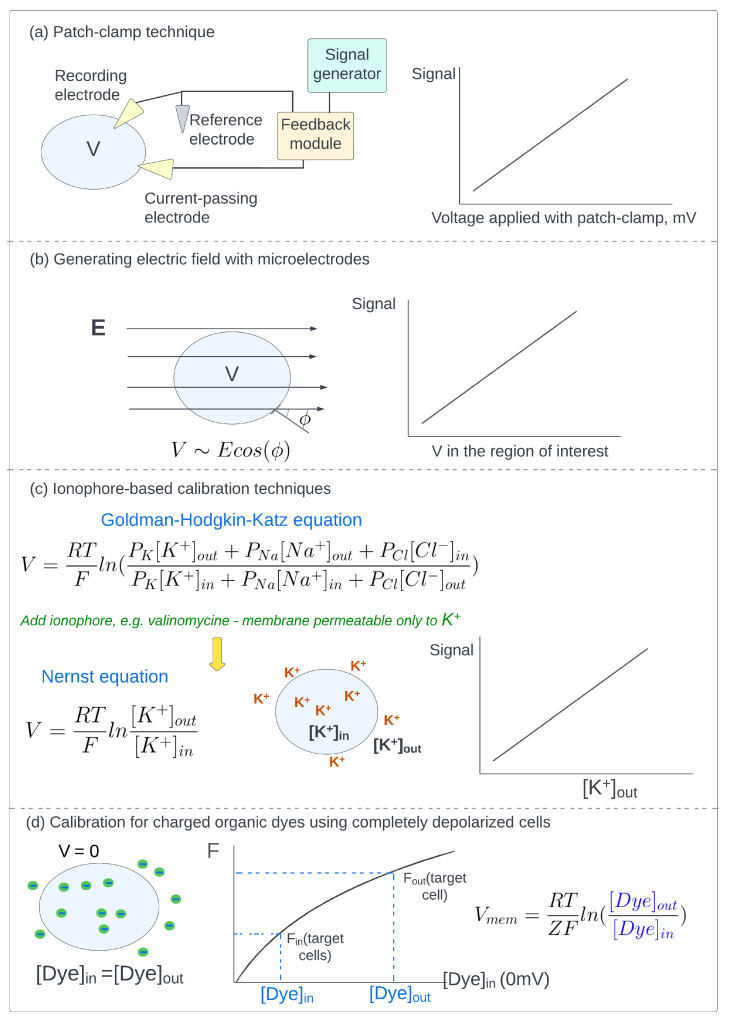
Methods used to calibrate sensors for membrane voltage imaging to measure optical signals for a series of established membrane voltage values. (**a**) Patch-clamp technique. The specific value of cell membrane potential can be established using the patch-clamp device. Actual cell membrane potential is measured as the voltage difference between the recording electrode patched to the cell membrane and the reference electrode placed into an electrolyte or extracellular medium. The value is passed to the feedback module, which determines the difference between the actual and desired values set by a signal generator. The difference is compensated by the injection of an electric current into the cell through the current-passing electrode. (**b**) Generating electric field with microelectrodes. A cell is placed between two microelectrodes that generate a uniform electric field *E*. The corresponding changes in cell membrane potential are proportional to the magnitude of the electric field and the cosine of the angle between the electric field vector and membrane surface. (**c**) Ionophore-based calibration techniques. The addition of valinomycin ionophore makes membrane voltage determined solely by the extracellular concentration of potassium ions. For calibration, fluorescence intensities are measured for a series of extracellular potassium concentrations. (**d**) Calibration of charged organic dyes using completely depolarized cells. For charged dyes, membrane voltage can be derived from the ratio of intracellular and extracellular concentrations in accordance with the Nernst equation. The relationship between detected fluorescence intensity and dye concentration can be derived by the measurement of fluorescence intensity for a set of extracellular dye concentrations using cells depolarized to 0 mV, when intracellular and extracellular concentrations are equal. Afterward, ${\left[Dye\right]}_{in}$ and ${\left[Dye\right]}_{out}$ can be obtained from the calibration plot.

The most direct way to establish desired membrane voltage value is to use a patch-clamp device (Figure 3a). Actual cell membrane voltage is measured as the voltage difference between the recording electrode patched to the cell membrane and the reference electrode placed into an electrolyte or extracellular medium [117]. The recorded value is provided as input for the feedback module, which compares it with the desired value set by the signal generator. To compensate for the difference between actual and desired membrane potential values, electric current is injected into the cell via the third, current-passing electrode. To obtain the calibration curve, optical signal is recorded for a series of voltage values, usually in the −100 mV to +100 mV range.

### 5.2. Generating Electric Field with Microelectrodes

Cells can be placed between microelectrodes [118] or in specific chambers [119] that generate a uniform electric field (Figure 3b). For a single spherical cell, membrane voltage changes induced by the field demonstrate angular distribution in accordance with Schwan’s Equation [120,121]:

(8) $$\Delta V_{mem}=3/2aEcos\left(\theta \right)$$

Here, *a* is the cell radius, *E* is the magnitude of the electric field, and θ is the angle between the electric field and the membrane surface. For groups of cells or non-spherical cells, the electric field induces a more complex distribution of membrane voltage changes [119]. However, the application of an external electric field was shown to cause non-negligible pore formation, and the observed voltage changes were smaller than those derived from Equation (8) [118].

### 5.3. Ionophore-Based Calibration Techniques

In a normal state the resting membrane potential of a cell is determined by unequal intracellular/extracellular concentrations and permeability of different ions in accordance with Goldman-Hodgkin-Katz Equation [122]:

(9) $$V_{mem}=\frac{RT}{F}ln\left(\frac{P_{K}{\left[K^{+}\right]}_{out}+P_{Na}{\left[Na^{+}\right]}_{out}+P_{Cl}{\left[Cl^{-}\right]}_{in}}{P_{K}{\left[K^{+}\right]}_{in}+P_{Na}{\left[Na^{+}\right]}_{in}+P_{Cl}{\left[Cl^{-}\right]}_{out}}\right)$$

Here, R is the universal gas constant, T is the absolute temperature, F is the Faraday constant, $P_{X}$ is the permeability of ion X, ${\left[X\right]}_{in}$ and ${\left[X\right]}_{out}$ are the intracellular and extracellular concentrations of ion X, respectively. The addition of specific ionophores makes the cell membrane predominantly permeable to a single ion type, therefore, resting membrane voltage can be estimated with the Nernst Equation (Equation (2)). Usually, valinomycin ionophore is applied to make membrane voltage determined solely by the extracellular concentration of potassium ions [71,123,124]:

(10) $$V_{mem}=\frac{RT}{F}ln\frac{{\left[K+\right]}_{out}}{{\left[K+\right]}_{in}}$$

If the intracellular potassium concentration is known or estimated, then calibration can be performed by measuring signal values for a series of extracellular ion concentrations, which are directly related to membrane voltage values in accordance with Equation (10) (Figure 3c). The protocol cannot be used for dyes that interact with ionophore [113,125,126], and the accuracy of calibration plot depends on the error in the estimation of intracellular concentration of potassium ions [113,127,128,129].

### 5.4. Calibration of Charged Organic Dyes Using Completely Depolarized Cells

For charged dyes, membrane voltage can be derived from the ratio of intracellular and extracellular concentrations in accordance with the Nernst Equation (Equation (2)). The calibration plot, i.e., the relationship between detected fluorescence intensity and dye concentration, can be obtained by the measurement of fluorescence intensity for a set of dye concentrations using cells depolarized to 0 mV, i.e., when intracellular and extracellular dye concentrations are equal [70,71]. After the calibration procedure, intracellular concentration ${\left[Dye\right]}_{in}$ can be obtained from the calibration curve as a value corresponding to fluorescence intensity detected from the cell interior of intact cells. Extracellular concentration ${\left[Dye\right]}_{out}$ can be obtained as a value corresponding to fluorescence intensity detected from the interior of completely depolarized cells (Figure 3d). The obtained values are taken to calculate membrane potential using the Nernst Equation [69,72].

## 6. Conclusions

Membrane potential imaging in combination with optogenetic and photopharmacological techniques [130,131,132,133,134,135,136,137,138,139,140,141,142] has been already successfully applied for the investigation of fast processes involving electrically excitable cells, such as neurons and cardiomyocytes. Further progress in the investigation of excitable cells and an effective extrapolation of the approach to other cell types requires improvement of both corresponding tools and techniques. One direction for improvement is the development of new potentiometric sensors with optimized properties—bright and photostable fluorescence, red-shifted absorption and emission bands, fast fluorescence response to voltage changes, a high magnitude of fractional fluorescence changes in response to voltage, etc. Another direction, which is especially important for the investigation of long-term processes and in vivo studies, is the further development of methodologies to eliminate optical signal changes related to factors other than voltage and methodologies to derive absolute cell membrane potential values. Examples of successful applications of such methodologies are still limited. Their further development can substantially increase the number of biological systems and processes that can be investigated with membrane potential imaging techniques and open a new dimension in various areas of biology and biomedicine.
